# Mitochondrial DNA Haplogroups and Susceptibility to Prostate Cancer in a Colombian Population

**DOI:** 10.1155/2014/530675

**Published:** 2014-01-28

**Authors:** D. Cano, C. F. Gomez, N. Ospina, J. A. Cajigas, H. Groot, R. E. Andrade, M. M. Torres

**Affiliations:** ^1^Human Genetics Laboratory, Science Faculty, Universidad de los Andes, Bogotá, Colombia; ^2^Prostate Clinic, Fundación Santa Fe de Bogotá University Hospital, Bogotá, Colombia; ^3^Urology Department, Hospital Militar Central, Bogotá, Colombia; ^4^Pathology and Laboratory Department, Fundación Santa Fe de Bogotá University Hospital, Bogotá, Colombia

## Abstract

Prostate cancer (PC) is one of the most common cancers and the second leading cause of mortality from cancer in Colombian men. Mitochondrial DNA (mtDNA) haplogroups have been associated with the risk of PC. Several studies have demonstrated dramatic differences regarding the risk of PC among men from different ethnic backgrounds. The present study was aimed at assessing the relationship between mtDNA haplogroups and PC. The mitochondrial DNA hypervariable segment I (HSV-1) was sequenced in a population-based study covering 168 cases (CA) and 140 unrelated healthy individuals as a control group (CG). A total of 92 different mtDNA sequences were found in CA and 59 were found in the CG. According to the geographical origin attributed to each mtDNA haplogroup, 82% of the mtDNA sequences found in both groups were Native Americans (A, B, C, and D). The most frequent was A (41.1%CA–42.1%CG), followed by B (22.0%CA–21.4%CG), C (12.0%CA–11.4%CG), and D (6%CA–10.0%CG). A lower percentage of European haplogroups (U, H, K, J, M, T, and HV) were also found (13.1%CA–12.9%CG), likewise African haplogroups (L0, L1, L2, and L3) (6.5%CA–2.1%CG). There were no statistically significant differences between the distribution of mtDNA haplogroups in CA and the CG in this study.

## 1. Introduction

The precise molecular events leading to prostate carcinogenesis are currently not well known. The genetic characterization of this neoplasm has mainly been focused on the nuclear genome, showing complex chromosomal instability as one of the main changes; however, the cause of the diversity of chromosomal alterations detected in patients is still unclear. The presence of mutations in oncogenes and tumor suppressor genes has been associated with late events in the progression of prostate cancer (PC) [1].

Mitochondrial DNA (mtDNA) is the main target for cellular reactive oxygen species (ROS) and it has been observed that the level of oxidative damage is more extensive and persistent in this than in the nuclear genome, thereby leading to the accumulation of greater numbers of mutations [2]. Recent studies have shown that the presence of multiple homoplasmic point mutations in the mitochondrial genome is common in many human tumors, such as those found in colon and prostate cancers [3, 4].

These mutations could also lead to mitochondrial dysfunction due to alteration of the intermediary metabolism, which could be interpreted as a signal for inducing tumor pathogenesis [5].

The location of these mutations within the genome has been correlated with different types of cancer. The presence of mutations in the cytochrome oxidase I (COI) gene occurs in around 11% of PC patients [6]. On the other hand, the noncoding displacement loop (D-loop) region proved to be a critical site for the presence of mutations (mutational hotspot) in neoplasm of the bladder, lung, head, and neck. These mutations are associated with the D-loop function as a regulatory site for this genome's replication and expression [7].

mtDNA is characterized by a strictly maternal mode of inheritance, the absence of recombination, a rapid mutation rate, and high level of population-specific polymorphisms. Mutation accumulation in mtDNA is tenfold greater than in nuclear DNA. This feature has created and characterized groups defined by having a maternal lineage legacy, making mtDNA a useful tool for studying origin and migration in human populations; it is widely applicable for studying evolutionary relationships among human ethnic groups [8]. The control region (D-loop) is the most variable region in the mitochondrial genome and the most polymorphic nucleotide sites are concentrated in two hypervariable segments (HVS-I and HSV-II). Individuals' geographical origin has been identified by high-resolution RFLP analysis and HSV-I sequencing [9–20].

Studying mtDNA haplogroups has been of great interest as this presents a potential disease susceptibility biomarker in different population groups. The growing number of publications describing the risk of cancer associated with various mtDNA haplogroups in the human population has challenged the reported mutations' validity and their use as susceptibility biomarkers [21–23]. Systematic errors are frequently reported in anthropological and forensic science studies because of the multiple steps involved in analyzing mtDNA sequences. More than half of the sequences contain obvious errors [24]. Identifying legitimate mtDNA mutations often becomes confused by a heteroplasmy event, a condition in which both wild and mutant genomes coexist within the same cell [25]. “Phantom” mutations (systematic errors or artifacts produced during sequencing) can also create a different mutation pattern from that produced in the cell in natural conditions [26]. These mutations have been called “innovative” by some authors and erroneous conclusions have led to a false interpretation of results regarding their association with different diseases [27, 28]. Some authors have suggested that this type of study should be analyzed in the light of haplogroup phylogeny, taking their diversification in younger clades and those having limited geographical and ethnic distribution into account, as well as identifying shared frequent mutations, to avoid such errors [9, 29].

Several studies have linked PC susceptibility to individuals' ethnic origin which could suggest a relationship between population variability and the genetics of cancer [30–32]. Previous research has shown that the incidence is two to ten times greater in North Americans of African descent when compared to Caucasians and Asians, respectively [33]. In this regard, studies by Booker et al. [34] have shown that haplogroup U (European origin) is associated with about twice the risk of PC and 2.5 times the risk of renal carcinoma in American individuals having European ancestry. Contrary to this finding, Kim et al. [35] did not reveal any association between Asian and PC lineages for the Korean population. Similarly, research by Muller et al. [36] found no association between these haplogroups and PC in a European population.

Ancestral studies in the Colombian population have been conducted in the past, focusing on determining the population's origin; however, very few works have been carried out to date which have focused on complex diseases such as PC. No study which has been carried out on admixed Latin-American populations has sought to associate maternal lineage (mtDNA haplogroup) with susceptibility to PC; this would be of great interest, as these admixed populations are historically shaped by three major human geographical groups: Native Americans, Europeans, and Africans. Bearing this in mind, the present study was aimed at establishing ethnic origin based on 168 sequences from the mtDNA hypervariable segment I (HSV-1) in a group of PC patients compared to 90 sequences from healthy patients, thus correlating frequency and possible disease susceptibility to one of the recognized mtDNA haplogroups.

## 2. Methods

### 2.1. Study Population

This work forms part of a case-control study seeking to identify PC susceptibility biomarkers. The study population therefore started from a group of 310 patients having a confirmed diagnosis of PC and who had undergone radical prostatectomy and 152 individuals who were seeking medical attention at the same medical centers (Fundación Santa Fe de Bogotá and Hospital Militar Central, Bogotá, Colombia) who did not have clinical and/or paraclinical PC (controls) and who were randomly selected (not related to the cases) from the same place of birth. 168 patients and 140 controls were selected from this group for analyzing mtDNA HSV-1 sequences. Clinical information was also available which took into account clinical-pathological variables such as prostate-specific antigen (PSA) level [37], tumor aggressiveness parameters and grade according to Gleason score [38], and tumor status (TMN), according to World Health Organization (WHO) recommendations. Likewise, a survey was carried out for identifying the origin of the studied specimens by family. The Universidad de los Andes' Research Ethics Committee had already approved a research proposal entitled “A search for genetic markers able to identify prostate cancer susceptible individuals.” All participants signed an informed consent form and the study was carried out in-line with the Declaration of Helsinki principles (2000).

### 2.2. DNA Isolation and Sequence Analysis

DNA was extracted from whole blood collected from each individual by the salting-out method. mtDNA sequences were analyzed from position 16046 to 16373 (a 328 bp fragment). PCR was performed with primers L15996 (CTCCACCATTAGCACCCAAAG) and H16401 (TGATTTCACGGATGGTG) for amplifying the mtDNA fragment. Each reaction was carried out in 25 *μ*L containing 12.5 *μ*L Master Mix, 1.25 *μ*L of each primer, 9 *μ*L distilled water, and 1 *μ*L DNA sample. Thermal cycle conditions were 95°C for 3 min, 35 cycles of 95°C for 1 min, 54°C for 1 min, 72°C for 1 min, and a final extension step at 72°C for 5 min. The mutations in the sequences were thoroughly reviewed to verify their existence using chromatograms and Geneious software [39]. MUSCLE multiple alignments (default parameters) were separately made with the patients and controls' sequences, using the consensus revised Cambridge reference sequence (CRS) [40]. Phylogenetic analysis was performed for determining relationships between the HSV-1 region sequences using a phylogenetic tree built with the Neighbor-Joining method using MEGA 4.0 software [41] with the Kimura-2P evolutionary model which led to organizing the haplogroups into distinct clades according to their mutations. A table was created in Excel (Microsoft Office 2007) showing the segregating sites for each haplotype and their location in the genome for better visualization of the specific mutations determining each haplogroup. Current references were used for assigning the haplogroups to each sequence for achieving a much more specific subhaplogroup classification. A haplotype network was constructed with Network 4.5 (Fluxus Technology) for visualizing phylogeny which showed haplogroup ramifications depending on case and control frequencies.

### 2.3. Data Analysis

mtDNA haplogroup frequencies were calculated by directly counting the observed phenotypes. A Chi-square test was made in a two-by-two table, between pairs of patient and control samples using PASW Statistics 18.0 software (SPSS GmbH Software, 80 339 Munich, Germany) to test whether the population had significant differentiation. The test's significance level was applied with <0.05 probability as cutoff. A proportions and odds ratios (OR) test was then made with 95% confidence interval.

## 3. Results

### 3.1. Population Characteristics

Table 1 gives a description of the characteristics of the population being studied. Mean age at onset was 68.02 (±9.18) years for PC patients and 62 (±12.0) for the control group. Regarding PSA range, only 22% of the patients had PSA levels below 4 (remaining normal), while the vast majority of patients had levels above normal, ranging from 4.1 to 10.0 ng/mL (49.4%), from 10.1 to 20.0 ng/mL (19.4%), and above 20.0 ng/mL (9.4%). Most of the control group had normal PSA levels (89.3%). The clinical and histological parameters regarding histological grade showed that 64.5% of the specimens had a Gleason score below 7 and 35.5% had a value greater than or equal to 7. Regarding tumor status (TMN), the vast majority of patients were in stages 1 and 2. The case-control population surveyed here was characterized by having a high frequency of individuals (90%) from Andean region departments, most from the Cundinamarca-Boyaca plateau, and less frequently from the Caribbean area and abroad.

### 3.2. Analyzing mtDNA Genetic Diversity in the Study Population

**Figure 1:**
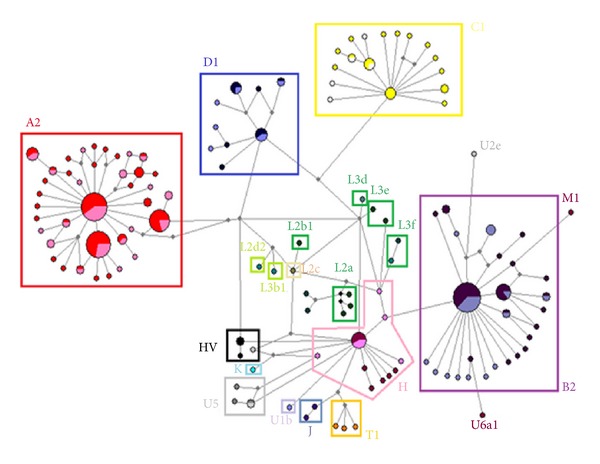
Network haplotype constructed by the Median-Joining method showing haplogroup distribution (light colors represent control patients, while dark ones represent cases).

Ninety-two different haplotypes were identified in the group of patients (168) which showed 90 polymorphic sites. Fifty-nine different haplotypes were found in the control group (140) in which 67 polymorphic sites were observed; 17 haplotypes were shared in the cases and 10 in the control group (Table 2). A haplotype network was constructed using the Median-Joining method (Figure 1) with all the haplotypes from both the patients and control group to establish relationships between HSV-1 region sequences and identify distinctive haplogroups according to their mutations shared by clades. The network arrangement showed four groups featuring Amerindian haplogroups, A2, B2, C1, and D1. European-origin sequences (U, H, HV, M, and T) were also found in a group more closely related to the revised reference sequence (CRS-Anderson). Some sequences had many mutations which generated outstanding long branches that were subsequently identified as belonging to African haplogroups (L). The different literature references [42] were used for assigning haplogroups to find their characteristic mutations; the diagnostic position for each haplogroup is shown in Table 2.

### 3.3. Association Studies of mtDNA Genetic Diversity between Cases and Controls

**Figure 2:**
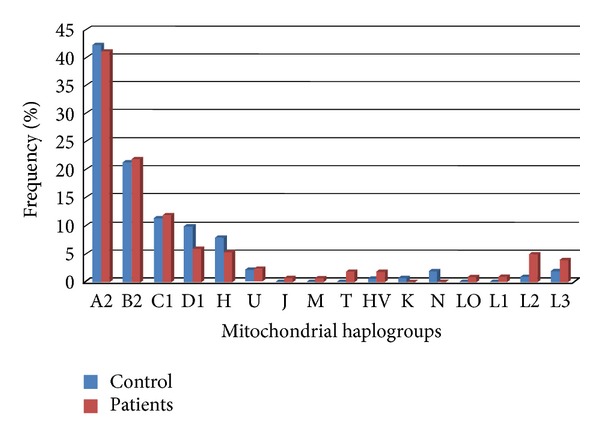
Histogram of mtDNA haplogroup frequency in the study population.

Figure 2 shows mtDNA haplogroup frequency distribution in Colombians. The population was characterized by Amerindian haplogroups, having high haplogroup A2 frequency in both patient and control groups (41.1% and 42.1%, resp.), followed by haplogroup B2 (22% cases and 21.4% controls); the other Amerindian haplogroups were also found to have moderate frequencies in the population. Previous mtDNA studies have shown that the Colombian population located in the Central and Andean areas has the same lineages which is consistent with our findings. Minor frequencies of European haplogroups (U, T, H, HV, and J) were found and haplogroup U (associated with prostate cancer by Booker et al., in North American individuals) was present in 2.4% of cases and 2.1% of controls. African haplogroups (L) were found in 6.5% of cases and 2.1% of controls. Our results had no statistically significant differences regarding the distribution of the mtDNA haplogroup frequencies between each haplogroup for case and control groups (Table 3). However, haplogroup L was more frequent in PC patients. Each mtDNA haplogroup was rearranged according to its Amerindian, European, and/or African origin and the association with PC was tested by using logistic regression analysis (Table 4).

**Figure 3:**
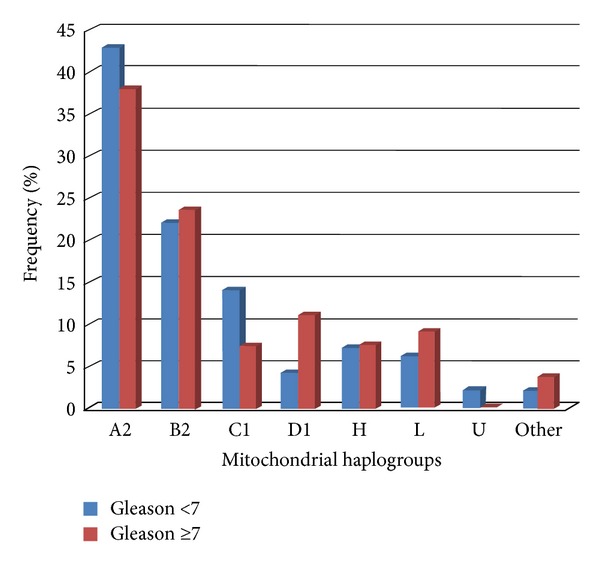
Histogram of mtDNA haplogroup frequency by Gleason score.

No statistically significant association was found between the presence of European or African origin and prostate cancer risk in the first analysis, using Amerindian ancestry as reference group; in the second analysis, using European ancestry as reference group, no statistical association was found between Amerindian ancestry and prostate cancer risk. Mitochondrial haplogroup frequencies did not differ significantly between patients having <7 Gleason score and patients having ≥7 Gleason score (Figure 3). No statistically significant association was observed when population origin was compared between prostate cancer patients having <7 Gleason score and patients having ≥7 Gleason score (Table 5).

## 4. Discussion

Besides being the major source of intracellular ATP, mitochondria perform other cellular functions related to cell growth, apoptosis-mediated cell death, and that mediated by other metabolic pathways. The role of mitochondria in carcinogenesis has also been documented, mainly regarding specific mitochondrial DNA polymorphism in genes involved in phosphorylation pathways. Its metabolism and function in oxidative phosphorylation play a crucial role in ROS production, causing DNA damage and increased cancer risk, resulting in an increased frequency of mutagenesis, in either mitochondrial or nuclear DNA. Recent studies have described an increased risk of cancer associated with a specific mitochondrial DNA haplogroup in the human population. Verma et al. have shown that subjects having the M7b2 haplogroup tended to have an increased risk for leukemia [43]. Polymorphisms in haplogroup N are associated with the risk of breast cancer in females from India [44]. Canter et al. found substitutions in ND3 associated with increased risk in African American females when studying patients with breast cancer in the USA [45]. mtDNA mutations in cancer patients frequently involve the regulatory D-loop region affecting different mitochondrial genes' copy numbers [46, 47]; mutations in other parts are also seen in patients suffering from specific types of cancer resulting in substantial changes in the expression of cellular proteins acting as tumor suppressor factors or oncogenes [48].

The aim of this case-control study, conducted on 168 men with PC and 140 normal subjects (both from a Colombian population), was to seek an association between the presence of any of the mtDNA haplogroups and susceptibility for suffering from PC (special emphasis was paid to the Amerindian haplogroups A, B, C, and D). No statistically significant differences were found between haplogroup distributions in prostate cancer patients compared to the control group in the population being studied.

The samples were genotyped and revealed a high frequency of Amerindian-origin mitochondria, which was corroborated by previously published studies about the origin of current populations in Colombia [13]. This study revealed the presence of two Amerindian haplogroups (A and B both with high frequency) which are the characteristic of the Colombian population belonging to the Chibcha-Paez linguistic family which is more common in the northern part of the country. Amerindians' important contribution to Latin Americans' mtDNA gene pool has been documented in a Colombian population, by contrast with paternal lineage shown by Y chromosome markers which revealed a high prevalence of European origin [49]. Likewise, the presence of lower percentages of African and European-origin haplogroups was observed in both cases and controls.

Previous studies have correlated the presence of a specific haplogroup with the risk of developing PC. Booker et al. [34] found mitochondrial haplogroup U overrepresentation in a group of PC patients compared to the control group (OR: 1.95). In another study, which tried to replicate these results, Canter et al. reported 26.7% of haplogroup U in their prostate cancer group (*n* = 71) and 11.7% for the control group (*n* = 128). However, their results were limited by the low number of patients involved. Additionally, the report failed to explain study participants' characteristics or the selection process, by contrast with our study, in which both patients and controls shared the same characteristics, such as geographical family background and coming from the same source (hospitals). Recent studies conducted on a European Caucasian population found no significant differences between haplogroups distribution when comparing PC patients (*n* = 304) to controls (*n* = 278) [36]. Moreover, consistent with our findings, another study in the Korean population found no association between mitochondrial haplogroups and PC risk [35]. A set of 22 East Asian haplogroups was discussed, finding no statistically significant difference in mtDNA haplogroup frequency distribution between cases (*n* = 139) and the control groups (*n* = 122).

There is great geographical variation in haplogroup frequencies between the above studies' populations and ours, especially in a South American country like Colombia, having great population diversity determined by the admixture of Indigenous, European, and African haplogroups, thereby revealing this study's importance. Amerindian mtDNA's high contribution was found in this study which revealed no association between population and PC risk, similar to studies carried out by Kim et al. [35] and Mueller et al., [36] which found no association of PC risk in their respective populations. To the best of our knowledge, no work relating PC susceptibility to haplogroups in Amerindian populations has ever been previously reported in the literature, making this a novel work and emphasizing its importance compared to other studies conducted on other populations. However, to fully understand population-based studies' importance and the risk of PC, more ancestry-informative markers (AIMs) should be taken into account to establish a correlation with susceptibility to this disease; mtDNA-related epidemiological studies are thus presenting a high prevalence of sequence errors due to incorrect processing during mutation review, leading to erroneous results and misinterpretation.

The importance of using appropriate control groups in such studies has been noted and, more importantly, the validity of known and novel mutations due to errors in sequencing or heteroplasmy was also known. Achilli et al. [9] proposed that association studies should be preformed, taking phylogenetic tools into account whish allow recognition of characteristic mtDNA polymorphic sites to be compared with those already established in several publications. The sequences were thoroughly reviewed in our study to verify that mutations were present; a phylogenetic tree was then constructed which helped to identify the segregating sites belonging to each haplogroup, which was allocated by using earlier mtDNA studies.

In accordance with the study by Mueller et al., [36], as only mitochondrial haplogroups and HSV-1 polymorphisms have been analyzed in the control region, it cannot be ruled out that polymorphisms in the mtDNA coding region which have not been included in this study may be associated with PC; some somatic mutations have been reported in a variety of cancers, including PC.

Several studies on mixed populations from Colombia's Andean region have found that the most frequent Amerindian haplogroups A and B arose from maternal lineages (37%–50% for haplogroup A and 25%–35% for haplogroup B) and, less frequently, so did haplogroups C and D, as well as European and African haplogroups [50, 51]. The frequencies obtained in the present study thus concur with such results. By contrast with the population base on this study, where the number of haplogroups was relatively small, larger sample sizes would be needed to provide adequate power for detecting associations when a large number of haplogroups are present in a sample, as reported by Muller and Kim in Europeans and Asians, respectively.

In conclusion, there was no association between mitochondrial haplogroups or HSV-1 region polymorphism with PC in a Colombian population.

## Figures and Tables

**Table 1:** Clinical-pathological characteristics of Colombian prostate cancer patients and the control group.

Characteristic	Category	PC patients (*n* = 168)	Controls (*n* = 140)
Mean (SD^1^) age		68.02 (9.18)	62.62 (11.97)
		*n* = 162	*n* = 133

Mean (SD^1^) PSA^2^		2.16 (0.87)	1.12 (0.39)
		*n* = 160	*n* = 140
	≤4	35 (21.9%)	125 (89.3%)
	4.1–10.0	79 (49.4%)	12 (8.6%)
	10.1–20.0	31 (19.4%)	3 (2.1%)
	>20	15 (9.3%)	0 (0.0%)

Gleason score		*n* = 155	—
	Gleason score < 7	100 (64.5%)	—
	Gleason score ≥ 7	55 (35.5%)	—

TMN		*n* = 146	—
	T1	54 (37.0%)	—
	T2	58 (39.7%)	—
	T3	30 (20.5%)	—
	T4	4 (2.8%)	—

^1^SD: standard deviation.

^2^PSA: prostate-specific antigen.

**Table 2:** Variable nucleotide positions for the HVS1 mtDNA sequences obtained and their frequency in cases and control groups.

Control				Cases			
Hap	Haplogroup	*n*	HSV-1 (16000+)	Hap	Haplogroup	*n*	HSV-1 (16000+)
1	H	3	CRS	1	H	3	CRS
2	H	1	271, 304	2	H	1	188G
3	H	1	243, 311	3	H	1	230, 256, 352
4	H	1	189	4	H	1	129, 316
5	H	1	189, 223	5	H	1	129, 257, 316
				6	H	1	244, 295
				7	J	1	069, 126
				8	J	1	069, 126, 278
6	HV	1	278, 311	9	HV	1	134, 362
7	K	1	224, 311	10	HV	2	362
8	U1b	1	104, 111, 249, 327	11	M1	1	129, 183C, 189, 311
9	U2e	1	051, 126C, 183C, 189, 362	12	T	1	126, 163, 186, 189N, 294
				13	T	1	126, 292, 294
				14	T	1	111, 126, 294, 304
10	U5a	1	192, 270	15	U5a	1	192, 270
				16	U5a	1	192, 256, 270
				17	U5b	1	189, 192, 243, 270, 311, 336
				18	U6a	1	172, 183C, 189, 219, 278, 295
				19	L0a1	1	129, 148, 168, 172, 187, 188G, 189, 214, 223, 230, 278, 293, 311, 320
				20	L1c1a	1	129, 187, 189, 223, 274, 278, 293, 294, 311, 360
11	L2d2	1	093, 111A, 145, 184, 223, 239, 278, 292, 355, 362	21	L2a	1	131, 189, 223, 225, 234, 278, 294, 309
				22	L2a	1	093, 189, 192, 223, 278, 294, 309
				23	L2a	1	189, 223, 230, 278, 294
				24	L2b1	1	114A, 129, 213, 223, 278, 354
				25	L2c	1	223, 278
12	L3b1	1	124, 223, 234, 278, 362	26	L3e1	1	189, 207, 223, 327
				27	L3e1a	1	185, 209, 223, 327
				28	L3d	1	111, 124, 223
13	L3f	1	189, 209, 223, 292, 311	29	L3f	1	209, 223, 292, 295, 311
14	B2	1	182C, 183C, 189, 217	30	B2	1	086, 182C, 183C, 189, 217
15	B2	1	086, 182C, 183C, 189, 217	31	B2	1	182C, 183C, 189, 217, 301, 304
16	B2	1	129, 183C, 189, 217, 283C	32	B2	2	182C, 183C, 189, 217, 234, 362
17	B2	1	183C, 189, 217, 261, 284	33	B2	6	182C, 183C, 189, 217
18	B2	1	183C, 189, 217, 266	34	B2	1	097, 098, 182C, 183C, 189, 217
19	B2	1	183C, 189, 217, 274	35	B2	1	097, 098, 183C, 189, 217
20	B2	1	183C, 189, 217, 324	36	B2	1	182C, 183C, 189, 217, 324
21	B2	1	183C, 189, 217, 278, 355	37	B2	1	183C, 189, 217, 324, 366
22	B2	1	183C, 189, 217, 270, 304	38	B2	1	183C, 189, 217, 324
23	B2	1	183C, 189, 217, 299	39	B2	1	145, 157, 182C, 183C, 189, 217, 294, 311
24	B2	9	183C, 189, 217	40	B2	1	183C, 189, 217, 311
25	B2	3	098, 106, 183C, 189, 217, 362	41	B2	1	183C, 189, 217, 256
26	B2	1	183C, 189, 217, 234, 362	42	B2	1	093, 183C, 189, 217
				43	B2	1	179, 183C, 189, 217
				44	B2	1	183C, 186, 189, 216, 217
				45	B2	15	183C, 189, 217
				46	B2	1	183C, 189, 217, 278
27	C1	1	051, 223, 298, 304, 311, 325, 327	47	C1	1	205G, 223, 298, 325, 327
28	C1	1	051, 223, 298, 325, 327	48	C1	1	223, 265, 298, 319, 325, 327
29	C1	1	051, 209, 223, 298, 325, 327	49	C1	2	155, 223, 298, 325, 327
30	C1	1	169, 223, 298, 325, 327	50	C1	1	223, 298, 325, 327
31	C1	1	192, 223, 298, 325, 327	51	C1	1	051, 172, 223, 298, 325, 327
				52	C1	3	051, 223, 298, 325, 327
				53	C1	1	051, 209, 223, 298, 300, 325, 327
				54	C1	1	051, 209, 223, 298, 325, 327
				55	C1	4	223, 298, 325, 327
				56	C1	1	223, 290, 298, 325, 327
				57	C1	1	086, 223, 298, 325, 327
				58	C1	1	093, 223, 298, 325, 327
				59	C1	1	223, 278, 298, 325, 327
				60	C1	1	223, 259, 298, 325, 327
32	D1	1	183C, 189, 223, 325, 362	61	D1	3	183C, 189, 223, 325, 362
33	D1	1	189, 223, 272, 325, 362	62	D1	1	129, 223, 274, 325, 362
34	D1	1	142, 207, 223, 325, 362	63	D1	1	129, 189, 223, 325, 362
35	D1	1	093, 142, 223, 325, 362	64	D1	1	126, 223, 254, 325, 362
36	D1	2	223, 325, 362	65	D1	1	142, 223, 325, 362
37	D1	1	129, 223, 274, 325, 362	66	D1	3	223, 325, 362
38	A2	2	223, 290, 293T, 319, 362	67	A2	11	223, 290, 319, 362
39	A2	1	223, 290, 319, 362	68	A2	1	092, 111, 223, 290, 319, 356, 362
40	A2	1	223, 258N, 290, 319, 362	69	A2	1	111, 223, 290, 319, 356, 362
41	A2	1	223, 266, 290, 319, 362	70	A2	1	111, 223, 266, 290, 319, 356, 362
42	A2	1	111, 223, 239, 290, 319, 362	71	A2	1	092, 111, 223, 290, 319, 360, 362
43	A2	2	111, 223, 239, 290, 311, 319, 362	72	A2	1	075, 111, 175, 223, 259, 290, 300, 319, 362
44	A2	1	111, 189, 223, 239, 290, 319, 362	73	A2	4	111, 175, 223, 259, 290, 300, 319, 362
45	A2	2	111, 129, 223, 256, 290, 319, 362	74	A2	1	111, 175, 223, 259, 290, 319, 362
46	A2	1	111, 223, 287, 290, 319, 362	75	A2	1	111, 129, 223, 290, 319, 362
47	A2	1	111, 223, 319, 360, 362	76	A2	1	111, 129, 172, 223, 290, 319, 362
48	A2	1	111, 223, 290, 319, 360, 362	77	A2	3	111, 129, 223, 256, 290, 319, 362
49	A2	1	111, 129, 223, 290, 319, 362	78	A2	1	111, 213, 223, 286, 290, 319, 362
50	A2	1	093, 111, 223, 290, 319, 356, 362	79	A2	15	111, 213, 223, 290, 319, 362
51	A2	1	111, 213, 223, 290, 311, 319, 362	80	A2	1	111, 186, 213, 223, 290, 319, 362
52	A2	6	111, 213, 223, 290, 319, 362	81	A2	1	111, 213, 223, 290, 311, 319, 362
53	A2	1	111, 213, 223, 290, 293, 319, 362	82	A2	1	111, 131, 209, 223, 290, 311, 319, 362
54	A2	1	111, 187, 223, 290, 319, 362	83	A2	1	111, 223, 261, 290, 311, 319, 362
55	A2	1	111, 172, 223, 290, 319, 362	84	A2	13	111, 223, 290, 319, 362
56	A2	2	111, 223, 259, 290, 300, 319, 362	85	A2	1	111, 223, 240T, 290, 319, 362
57	A2	1	075, 111, 175, 223, 259, 290, 300, 319, 362	86	A2	1	093, 111, 223, 290, 299, 319, 362
58	A2	1	075, 111, 223, 259, 290, 300, 319, 362	87	A2	2	111, 223, 290, 319, 360, 362
59	A2	10	111, 223, 290, 319, 362	88	A2	1	086, 111, 171, 223, 290, 319, 362
				89	A2	1	111, 223, 290, 292A, 319, 362
				90	A2	2	111, 223, 290, 319, 355, 362
				91	A2	1	111, 197A, 223, 290, 319, 362
				92	A2	1	111, 223, 239, 290, 319, 362

**Table 3:** Frequencies (%) of mitochondrial DNA haplogroups in patients and controls.

Haplogroup	Patients with prostate cancer *n* = 168	Control group *n* = 140	*P* value^1^
A	41.1 (*n* = 69)	42.1 (*n* = 59)	0.84
B	22.0 (*n* = 37)	21.4 (*n* = 30)	0.89
C	11.9 (*n* = 20)	11.4 (*n* = 16)	0.89
D	6.0 (*n* = 10)	10.0 (*n* = 14)	0.18
H	5.5 (*n* = 9)	8.0 (*n* = 11)	0.37
HV	1.9 (*n* = 3)	0.8 (*n* = 1)	0.40
L	6.6 (*n* = 11)	2.1 (*n* = 3)	0.06
J	0.7 (*n* = 1)	0.0 (*n* = 0)	0.36
K	0.0 (*n* = 0)	0.7 (*n* = 1)	0.27
M	0.6 (*n* = 1)	0.0 (*n* = 0)	0.36
N	0.0 (*n* = 0)	1.4 (*n* = 2)	0.12
T	1.9 (*n* = 3)	0.0 (*n* = 0)	0.11
U	2.4 (*n* = 4)	2.1 (*n* = 3)	0.88

^1^ *P* value: Pearson chi-square or Fisher's exact tests.

**Table 4:** Association between prostate cancer risk and the population ancestry.

Ancestry*	Control	Prostate cancer	Odds ratio (95% CI)	*P* value	**Odds ratio (95% CI)	*P* value
Amerind	119 (85%)	135 (80.4%)	Reference		Reference	
European	18 (12.9%)	22 (13.1%)	1.08 (0.55–2.11)	0.82	1.09 (0.43–2.78)	0.85
African	3 (2.1%)	11 (6.5%)	3.23 (0.88–11.85)	0.07	1.01 (0.18–5.61)	0.99
European	18 (12.9%)	22 (13.1%)	Reference		Reference	
Amerind	119 (85%)	135 (80.4%)	0.93 (0.48–1.81)	0.52	0.91 (0.36–2.32)	0.85
African	3 (2.1%)	11 (6.5%)	3 (0.73–12.41)	0.12	0.35 (0.02–5.18)	0.44

*Analysis of ancestry was assessed by the use of the major mtDNA haplogroups.

**Adjusted by age, history of cancer, and PSA.

**Table 5:** Association between PC aggressiveness and ancestry of the patients according to the major haplogroups.

Population ancestry*	Gleason score < 7	Gleason score ≥ 7	Odds ratio (95% CI)	*P* value	**Odds ratio (95% CI)	*P* value
Amerindian	83 (83%)	43 (78.2%)	Reference		Reference	
European	11 (11%)	7 (12.7%)	1.23 (0.44–3.39)	0.69	1.13 (0.35–3.63)	0.83
African	6 (6%)	5 (9.1%)	1.61 (0.46–5.57)	0.45	1.36 (0.33–5.69)	0.67
European	11 (11%)	7 (12.7%)	Reference		Reference	
Amerindian	83 (83%)	43 (78.2%)	0.81 (0.29–2.25)	0.69	0.88 (0.28–2.83)	0.83
African	6 (6%)	5 (9.1%)	1.31 (0.29–5.98)	0.72	2.27 (0.27–19.38)	0.45

*Analysis of ancestry in patients was assessed by the use of the major mtDNA haplogroups.

**Adjusted by age, history of cancer, and PSA.
